# Transcriptomic Changes Due to Cytoplasmic TDP-43 Expression Reveal Dysregulation of Histone Transcripts and Nuclear Chromatin

**DOI:** 10.1371/journal.pone.0141836

**Published:** 2015-10-28

**Authors:** Alexandre Amlie-Wolf, Paul Ryvkin, Rui Tong, Isabelle Dragomir, EunRan Suh, Yan Xu, Vivianna M. Van Deerlin, Brian D. Gregory, Linda K. Kwong, John Q. Trojanowski, Virginia M.-Y. Lee, Li-San Wang, Edward B. Lee

**Affiliations:** 1 Department of Pathology and Laboratory Medicine, Perelman School of Medicine, University of Pennsylvania, Philadelphia, PA, United States of America; 2 Department of Biology, School of Arts and Sciences, University of Pennsylvania, Philadelphia, PA, United States of America; International Centre for Genetic Engineering and Biotechnology, ITALY

## Abstract

TAR DNA-binding protein 43 (TDP-43) is normally a nuclear RNA-binding protein that exhibits a range of functions including regulation of alternative splicing, RNA trafficking, and RNA stability. However, in amyotrophic lateral sclerosis (ALS) and frontotemporal lobar degeneration with TDP-43 inclusions (FTLD-TDP), TDP-43 is abnormally phosphorylated, ubiquitinated, and cleaved, and is mislocalized to the cytoplasm where it forms distinctive aggregates. We previously developed a mouse model expressing human TDP-43 with a mutation in its nuclear localization signal (ΔNLS-hTDP-43) so that the protein preferentially localizes to the cytoplasm. These mice did not exhibit a significant number of cytoplasmic aggregates, but did display dramatic changes in gene expression as measured by microarray, suggesting that cytoplasmic TDP-43 may be associated with a toxic gain-of-function. Here, we analyze new RNA-sequencing data from the ΔNLS-hTDP-43 mouse model, together with published RNA-sequencing data obtained previously from TDP-43 antisense oligonucleotide (ASO) knockdown mice to investigate further the dysregulation of gene expression in the ΔNLS model. This analysis reveals that the transcriptomic effects of the overexpression of the ΔNLS-hTDP-43 transgene are likely due to a gain of cytoplasmic function. Moreover, cytoplasmic TDP-43 expression alters transcripts that regulate chromatin assembly, the nucleolus, lysosomal function, and histone 3’ untranslated region (UTR) processing. These transcriptomic alterations correlate with observed histologic abnormalities in heterochromatin structure and nuclear size in transgenic mouse and human brains.

## Introduction

TDP-43 is a member of the heterogeneous ribonuclear protein group and binds to both DNA and RNA [1,2]. It has a range of functions that affect gene expression including regulation of transcription, alternative splicing, microRNA biogenesis, and RNA transport and translation (reviewed in [3–5]). Under normal physiological conditions, TDP-43 is predominantly localized in the nucleus [6], but is synthesized in the cytoplasm [7] and is shuttled between the nucleus and cytoplasm in a transcription dependent manner [8]. Several neurodegenerative diseases display TDP-43 pathology [4], and TDP-43 was identified as the main component of the distinctive cytoplasmic aggregates seen in ALS and FTLD-TDP [9]. In the pathological state, TDP-43 is cleared from the nucleus and mislocalized in the cytoplasm or neurites, in addition to being hyperphosphorylated, ubiquitinated, and cleaved [9–11].

Both a cytoplasmic gain of function and a nuclear loss of function of TDP-43 are possible disease mechanisms, as neurons with cytoplasmic TDP-43 aggregates are also associated with a loss of normal nuclear TDP-43 protein [9,12]. We previously [13] developed a tet-off inducible transgenic mouse overexpressing human TDP-43 (hTDP-43) with a mutated (K82A/R83A/K84A) nuclear localization signal (ΔNLS-hTDP-43) so that the protein preferentially localizes to the cytoplasm [7]. Despite the mainly cytoplasmic mislocalization of the hTDP-43 protein product, these mice did not exhibit a significant number of cytoplasmic aggregates. Interestingly, there was a time-dependent neurodegeneration which coincided with dramatic changes in gene expression as measured by microarray. An intriguing aspect of the observed gene expression effects was a selective and significant upregulation of chromatin assembly and histone genes, hinting at a role for TDP-43 in transcription and histone transcript stability.

In this study, we performed RNA-sequencing of ΔNLS-hTDP-43 transgenic mice brains to further study the transcriptomic effects of the gain of cytoplasmic TDP-43 in this model. This revealed significant changes in multiple molecular pathways, including confirmation that cytoplasmic TDP-43 expression alters transcription related pathways and histone transcript levels. Supporting a gain of cytoplasmic function mechanism, biochemical analysis of nuclear and cytosolic fractions of the ΔNLS-hTDP-43 mice brains showed that total nuclear TDP-43 expression is largely unaltered, suggesting that the exogenous human TDP-43 is able to compensate for autoregulatory reduction of endogenous nuclear mTDP-43. The transcriptomic changes were compared with RNA sequencing data from mice with reduced levels of TDP-43 after injection with an ASO against TDP-43 [14], revealing a complete absence of correlation between the two experiments, showing that the main effects of overexpressing ΔNLS-hTDP-43 are likely not due to the loss of TDP-43 function. Furthermore, we identify dysregulation of the histone 3’ end processing machinery upon transgene overexpression, identifying a mechanism by which overexpression of the ΔNLS-hTDP-43 transgene impairs the transcriptional machinery.

## Materials and Methods

### Ethics Statement

Animal care and performed procedures were in accordance with the NIH Guide for the Care and Use of Experimental Animals and approved by the University of Pennsylvania Institutional Animal Care and Use Committee (Permit Number: 803385).

### RNA sequencing of transgenic mice

Inducible transgenic mice overexpressing mutant ΔNLS-hTDP-43 were generated as described previously [13]. Briefly, a Camk2α promoter was used to drive expression of the tetracycline transactivator protein (Camk2α-tTa), and a tetracycline responsive promoter was used to drive ΔNLS-hTDP-43 expression (tetO-ΔNLS-hTDP-43) such that ΔNLS-hTDP-43 protein expression was inhibited by doxycycline in bigenic mice (Camk2a-tTa x tetO-ΔNLS-hTDP-43). Expression was repressed in bigenic mice (n = 4, 3 male) with a doxycycline diet until 28 days of age at which time expression was induced by removal of the doxycycline diet. RNA was obtained 10 days later because this is when neurodegeneration began in these mice [13]. Nontransgenic mice (n = 4, 3 male) bred from heterozygous parents for Camk2α-tTa and ΔNLS-hTDP-43 were used as controls, and followed the same diet timeline to control for doxycycline effects. RNA-seq libraries were constructed and sequenced as previously described [15], with mean RNA integrity number (RIN) 8.74. After mouse sacrifice, the right cortex was extracted and solubilized in TRIzol and treated with DNase. Polyadenylated messenger RNA was selected using the Dynabeads mRNA direct kit (Invitrogen), followed by fragmentation, 5’ phosphate addition, size selection, Illumina v1.5 RNA adapter ligation, cDNA library generation, PCR amplification, and purification before sequencing on an Illumina Genome Analyzer II. Raw reads and processed data have been deposited in NCBI’s Gene Expression Omnibus repository [16] and are accessible through GEO Series accession number GSE65973.

### Processing of RNA-seq data

Raw reads from both RNAseq experiments had their 3’ Illumina v1.5 smRNA adaptors (ATCTCGTATGCCGTCTTCTGCTTG) trimmed using cutadapt [17], were mapped to the mm9 *Mus musculus* genome build using Bowtie (version 0.12.7 with options—time -k 100—best—strata -v 2 -p 3) [18] and were further filtered to a maximum mismatch rate of 0.06. Only uniquely mapping reads were considered for further analysis except for histone analysis (see below). Coverage of the protein-coding segment of the genome was computed using the bedtools coverage [19] tool against the RefSeq mm9 genome, and coverage of the entire genome was computed using the bedtools genomecov [19] tool. The mapped reads from the RNA sequencing pipeline were analyzed for differential expression by using bedtools multicov [19] to compute the number of reads mapping to each exon. Exon counts were merged into gene counts using bedtools groupBy [19], which were then analyzed for differential expression with the DESeq2 R package [20]. Splicing analysis was performed using the DEXSeq R package [21] following the protocol described in the paper and vignette. Briefly, exon reads were counted using the dexseq_count.py script and read into R [22], where the DEXSeq analysis pipeline was followed using the Ensembl mm9 genome annotation [23]. For both the differential expression and the differential splicing analyses, significantly differentially expressed genes/exons were defined as having an adjusted p-value of less than 0.05 using the Benjamini-Hochberg multiple testing correction [24].

For pathway analyses, lists of refSeq IDs were obtained through R analysis and uploaded to NIH DAVID [25,26] for analysis. Background gene lists were the sets of genes meeting the minimum read count threshold (10 reads per million in at least one sample) in the relevant experiment being analyzed. From DAVID, lists of gene clusters and gene charts were obtained, using GOTERM_BP_FAT, GOTERM_CC_FAT, GOTERM_MF_FAT, and KEGG_PATHWAY categories for analysis. To reduce redundancy, these results were passed to a Ruby script, available from the authors, to group functionally similar pathways together into clusters. From each cluster of redundant pathways, the pathway with the lowest EASE p-value was selected for final analysis in R. To compute pathway fold changes, the refSeq IDs of genes in each pathway were matched with those from the DESeq2 results.

Previously published microarray data [13] is available under GEO accession number GSE25182. RNA sequencing data was compared with microarray data by finding matching probes by RefSeq ID.

### Analysis of HITS-CLIP data

The processed peaks from the CLIP sequencing data were directly obtained from the authors, as analyzed in [27]. A custom Python script (genewise_bed_coverage.py) was used to compute the number of CLIP hits and proportion of coverage of the CLIP hits over each genetic feature (introns, exons, 3’ untranslated region, and 5’ untranslated region). This script is available at https://github.com/alexamlie/bed_statistics.

### Bioinformatic Analysis of Histone Transcripts

To account for the fact that histones are grouped in highly repetitive clusters, we remapped and allowed for up to 10 multimappings for the 50 canonical and variant histone genes of interest, weighting each read as counting for *1/n* reads in each of its mapped locations, where *n* is the number of times that read mapped. To normalize, we chose 50 random unchanging genes, defined as having an adjusted DESeq2 p-value of greater than 0.5, and mapped them with up to 10 multimappings as well. Then, we took the geometric mean of the unchanging gene coverage for each sample, and converted these means into normalization factors by dividing them all by the mean of the geometric means of the nontransgenic samples. All histone genes with no coverage were converted to 0.1, the lowest possible coverage, and histone values were divided by the normalization factors. Finally, we took the separate geometric mean in each sample for the normalized canonical and variant histone genes. The changes in canonical and variant histones were quantified with 2-sided t-tests between the 4 bigenic and 4 nontransgenic normalized geometric means.

### Biochemical Analysis of Nuclear and Cytoplasmic Brain Lysates

Immediately upon sacrifice, cortices from mouse brains were dissected, weighed and frozen at -80°C. Total vs. nuclear vs. cytosolic fractions were prepared from cortex tissue as follows. Tissue was homogenized using 8 strokes of tight-fitted (B) Dounce homogenizer at 4°C in hypotonic buffer (10 mM KCl, 1.5 mM MgCl_2_, 1 mM DTT, 10 mM HEPES-KOH, pH 7.4 with protease inhibitors; 5:1 v/w). After a 10 minute incubation at 4°C, 1.0 M sucrose in hypotonic buffer (0.36:1 v/w) was added, and samples were Dounce homogenized with an additional 15 strokes. Aliquots were removed as the total fraction. Homogenates were then centrifuged at 1,000xg for 10 minutes at 4°C. 120 μL of the supernatant was taken and spun at 100,000xg for 30 minutes at 4°C. The supernatant from this spin represented the cytosolic fraction. The remaining supernatant and pellet from the 1,000xg spin were then taken, and 1.77 volumes of 2.3 M sucrose in TKM (50 mM Tris-HCl, pH 7.5, 25 mM KCl, 5 mM MgCl_2_) was added to raise the homogenate to 1.6 M sucrose. Additional 1.6M sucrose in TKM was then added to obtain ~4 mL of homogenate, which was overlaid on 3 mL of 1.8M sucrose in TKM followed by ultracentrifugation at 100,000xg for one hour at 4°C. The pellets were resuspended in 30 μL of PBS with protease inhibitors, and nuclei were counted using a hemocytometer. Finally, based on the nuclei counts, nuclei were diluted in PBS with protease inhibitors so that all samples contained the same concentration of nuclei. Sample buffer was added to all fractions followed by sonication and freezing at -80°C until SDS-PAGE analysis. Equal volumes of lysate were analyzed by electrophoresis on a 10–17% discontinuous step gradient tris-glycine polyacrylamide gel followed by transfer to nitrocellulose.

### Quantitative RT-PCR

RNA was extracted as described previously [13] from the same set of mice that were sequenced. Additional mice included bigenic mice maintained on doxycycline (n = 5, 2 male) and nontransgenic mice maintained on doxycycline (n = 5, 1 male). cDNA was generated using the SuperScript III First-Strand Synthesis System (Life Technologies) primed with either random hexamers or oligo-dTs for total RNA or polyadenylated RNA, respectively. qRT-PCR was performed on the cDNA using an Applied Biosystems 7500 Fast Real-Time PCR system using custom-designed primers (see S1 Table for primer sequences) and Power SYBR Green PCR Master Mix (Life Technologies). Gene expression values were determined by using the comparative C_T_ method. The genes of interest were standardized to the geometric mean of 2 housekeeping genes (*Gapdh* and *Actb*).

### Immunoblotting

The antibodies used for immunoblotting were rabbit polyclonal C1038 antibody against total TDP-43 [28], rabbit monoclonal anti histone H3 (Cell Signaling #4499P, Danvers, MA), and rat monoclonal anti HSP90 (Enzo 16F1, Farmingdale, NY). Immunoblots were visualized using an Oddysey Sa Infrared Imaging System (LiCor, Lincoln, NE) after application of fluorescent secondary antibodies (LiCor).

### Histology

Mouse brains and human brain tissue were fixed and processed into 6 μm sections as previously described [29]. To study nuclear chromatin, sections were stained with cresyl violet using standard methods. Human tissue sections were stained with anti-TDP-43 antibodies (C1039, [28]) followed by Alexa568-conjugated secondary antibodies (Life Technologies, Carlsbad, CA). DAPI was used as a nuclear counterstain. Sections were visualized with a Leica TCS SPE-II scanning laser confocal microscope to obtain z-stacks through the entire tissue section. Nuclear cross-sectional area was measured using Leica LAS AF software.

## Results

### RNA Sequencing of ΔNLS-TDP-43 Mice

**Fig 1:**
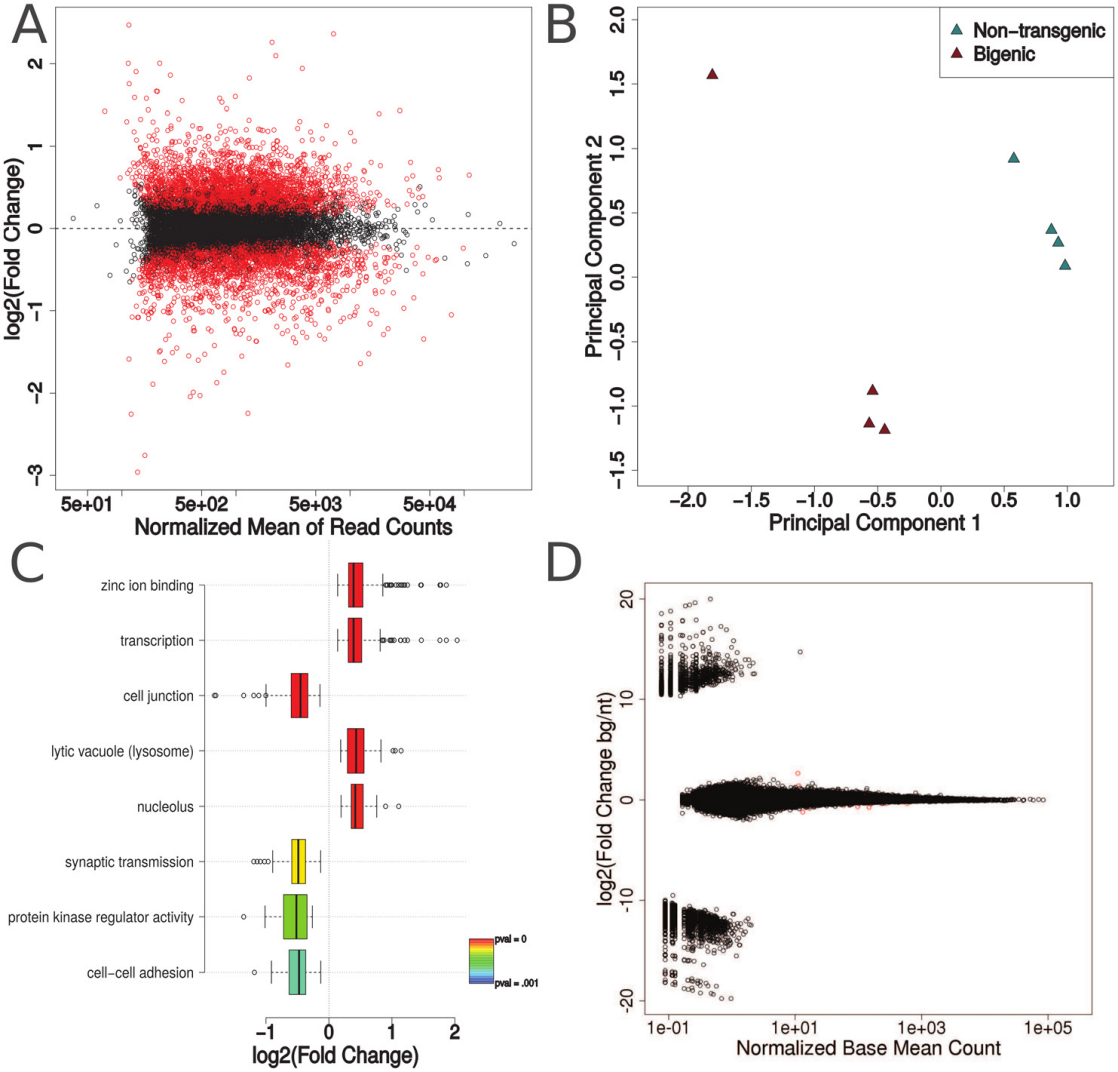
Transcriptomic analysis of ΔNLS-hTDP-43 expressing mice. (A), Comparison of normalized mean of read counts with log_2_(fold change) shows no bias for highly expressed genes to be categorized as differentially expressed. 4,321 out of 10,601 genes meeting the minimum read count threshold were found to be significantly differentially expressed in ΔNLS-hTDP-43 expressing mice compared to nontransgenic mice (Benjamini-Hochberg corrected p-value < 0.05, red points). (B), Multidimensional scaling and principal component analysis shows distinct global transcriptomic profiles for ΔNLS-hTDP-43 bigenic (n = 4) and control nontransgenic (n = 4) mice. (C), Pathway analysis of significantly changing genes between bigenic and control mice reveals an upregulation in pathways predominantly involved in nuclear molecular processes and a downregulation in pathways relating to neuronal or glial function. (D), Splicing analysis reveals that only 23 out of 250,620 exons (red dots) are significantly differentially expressed with Benjamini-Hochberg correct p-values of less than 0.05.

To further investigate the widespread transcriptomic changes previously observed by microarray [13], we performed poly(A)^+^-selected RNA sequencing on right cerebral cortex from bigenic mice (n = 4, 3 male) and nontransgenic (n = 4, 3 male) control mice. This yielded mean values of 51,551,711 raw reads and 31,146,050 uniquely mapped reads per sample, with an average of 1.262x coverage of the protein-coding segment of the genome and 0.496x coverage of the entire genome. Differentially expressed genes were identified using DESeq2 [20], which identified 4,321 out of 10,601 genes (*Mus musculus* mm9 genome annotation) meeting a minimum read threshold of 10 reads per million in at least one sample as being significantly differentially expressed. Of these genes, 2,166 were upregulated in the ΔNLS mice compared to the control mice, and the remaining 2,155 were downregulated (Fig 1a). By principal component analysis, the bigenic mice and the nontransgenic mice overall clustered separately, indicating a robust change in the transcriptome due to overexpression of ΔNLS-hTDP-43 (Fig 1b). Comparison of these results with the previous microarray analysis [13] found that 7,696 of the 10,601 genes meeting the minimum read threshold had matching probes in the microarray, including just 3,092 of the 4,321 significantly differentially expressed genes, illustrating the increased sensitivity of RNA sequencing over microarray technology. Of the 3,092 differentially expressed genes present in the microarray, 2,863 had at least one microarray probe changing in the same direction as that identified by RNA sequencing, supporting the validity of our RNA sequencing results.

To identify the effects on biological processes associated with the overexpression of cytoplasmically mislocalized ΔNLS-hTDP-43, pathway analysis was performed on the significantly up- and down-regulated genes in the bigenic mice as compared to nontransgenic controls using NIH DAVID [25,26]. This analysis revealed 8 significantly enriched (EASE score P-value < 0.001) pathways, 4 of which were up-regulated and 4 of which were down-regulated (Fig 1c). The up-regulated pathways included zinc ion binding, transcription, lytic vacuole, and nucleolus. Given that the zinc ion pathway contains several zinc finger binding proteins which are important in regulating transcription, these four pathways largely coincide with pathways hypothesized to play a central role in ALS and FTLD-TDP, namely RNA regulation and proteostasis [5].

In contrast, the down-regulated pathways, including synaptic transmission, cell-cell adhesion, cell junction, and protein kinase regulator activity, appear to represent processes involved in more general cellular activity such as cell-cell interactions. Thus, there is a possibility that these down-regulated pathways may reflect changes secondary to neuronal death and the disruption of neuropil due to reactive gliosis, consistent with the previously described beginnings of neurodegeneration observed in the ΔNLS-hTDP-43 expressing mice at this timepoint [13].

### Lack of splicing changes in NLS mice

Several reports have implicated TDP-43 in regulation of splicing activity [5,14,30]. To determine whether mice overexpressing ΔNLS-hTDP-43 show splicing changes, we used DEXSeq [21] to search for differentially spliced exons (Fig 1d). Out of 250,620 exons analyzed, only 23 were identified as differentially spliced (S2 Table). Manual inspection of the 16 unique annotated, non-hypothetical genes in this set revealed that these genes fall into two general categories. Several transcripts regulate transcription and RNA processing, including *Srsf5*, which is part of the serine/arginine rich family of proteins that are involved in splicing as well as other aspects of gene expression [31], *Med20*, an essential component of the Mediator complex that regulates transcription [32], and *Usp49*, a histone H2B deubiquitinase which has been shown to regulate splicing [33]. Other transcripts are involved in cell signaling and membrane activity, notably *Nrcam*, a cell surface molecule that has been associated with autism [34,35]. However, given that TDP-43 has known effects on RNA splicing within the nucleus, the relative paucity of splicing alterations observed here suggest that the main transcriptomic effects of ΔNLS-hTDP-43 overexpression may be due to a gain of cytoplasmic function and not a loss of nuclear splicing function. Alternatively, TDP-43 has several other RNA functions other than splicing which were not directly assessed here.

### Biochemical analysis of nuclear and cytoplasmic TDP-43 levels

**Fig 2:**
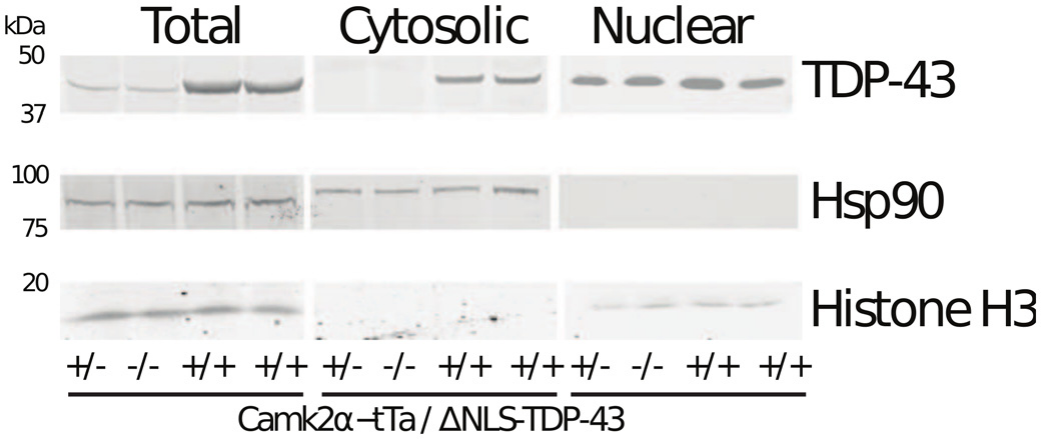
Nuclear and cytosolic fractionation of ΔNLS-hTDP-43 expressing mice. Subcellular fractionation followed by immunoblot protein analysis reveals a similar level of nuclear total (human and mouse, h+m) TDP-43 levels between bigenic (+/+) and control (+/- or -/-) mice, but an increase in cytosolic TDP-43 levels in the bigenic mice. HSP90 and histone H3 confirms separation of the cytosolic and nuclear fractions, respectively. n = 4 mice per category (4 bigenic, 2 monogenic, 2 non-transgenic).

Expression of ΔNLS-hTDP-43 leads to predominantly cytoplasmic transgene expression [7], and using species-specific TDP-43 antibodies, we have previously shown that ΔNLS-hTDP-43 expression is associated with a reduction of endogenous nuclear mTDP-43 protein [13]. The relative absence of splicing alterations in ΔNLS-hTDP-43 mice suggests that at least some TDP-43 protein was present within the nucleus, resulting in functional recovery from the loss of mTDP-43 in terms of splicing and supporting the idea that the effects of transgene overexpression are mainly due to cytoplasmic gain of function. To biochemically investigate the levels of TDP-43 protein in ΔNLS-hTDP-43 expressing mice, we performed nuclear and cytosolic fractionation on neocortex from bigenic (Camk2a-tTa x tetO-ΔNLS-hTDP-43) and non-transgenic or monogenic control mice. Samples were immunoblotted for total (mouse and human) TDP-43 protein. As expected, the bigenic mice showed increased cytosolic levels of total TDP-43 (Fig 2). Moreover, total nuclear TDP-43 protein was slightly higher in ΔNLS-hTDP-43 mice relative to control mice, suggesting that the exogenous hTDP-43 is indeed able to localize to the nucleus despite the mutation in the NLS. This slight nuclear increase may also contribute to toxicity. The lack of histone H3 in the cytosolic fraction and the lack of HSP90 in the nuclear fraction confirmed successful fractionation. These results demonstrate that ΔNLS-hTDP-43 overexpression leads to a marked increase in cytoplasmic TDP-43 protein with relative preservation of total nuclear TDP-43 protein levels, despite the loss of endogenous mTDP-43. The increased presence of cytoplasmic TDP-43 protein supports a gain of cytoplasmic function as one potential mechanism underlying the observed transcriptomic changes, but additional mechanistic studies to demonstrate this functionally are needed.

### Comparison with ASO TDP-43 Knockdown Gene Expression Data

To further characterize the transcriptomic effects specific to the overexpression of ΔNLS-hTDP-43, we compared our RNA-sequencing results with published RNA-sequencing data from mice injected with an ASO against TDP-43 which reduces endogenous mTDP-43 protein levels [14]. While the differentially expressed genes in ΔNLS-hTDP-43 mice seem to be mainly caused by a cytoplasmic gain of exogenous TDP-43 function as shown by Western blot analysis and the lack of splicing changes, the differentially expressed genes upon ASO knockdown are more likely to be directly linked to the loss of normal, endogenous TDP-43 protein. Thus, if the main effects in the ΔNLS-hTDP-43 model are indeed caused by a cytoplasmic gain of function, comparison of the transcriptomes of these two mouse models should show different effects.

Raw sequencing reads from the ASO experiment (GEO database accession number GSE27218), were analyzed with the same pipeline used for ΔNLS-hTDP-43 mice. Technical replicates were combined to analyze biological replicates of control ASO injected mice (n = 4, 4 female) versus TDP-43 ASO injected knockdown mice (n = 4, 4 female). Across these samples, we found a mean of 30,794,457 total reads, with a mean of 29,132,557 uniquely mapping. We were able to map more reads than the original study [14], which used an older version of Bowtie (0.12.2) and only uniquely mapped about 50% of total reads, compared to the newer version we used (0.12.7), reflecting advances in the accuracy of Bowtie. These datasets demonstrated mean coverage of 1.580x over the protein coding segment of the genome, and a mean coverage of 0.599x over the entire genome.

**Fig 3:**
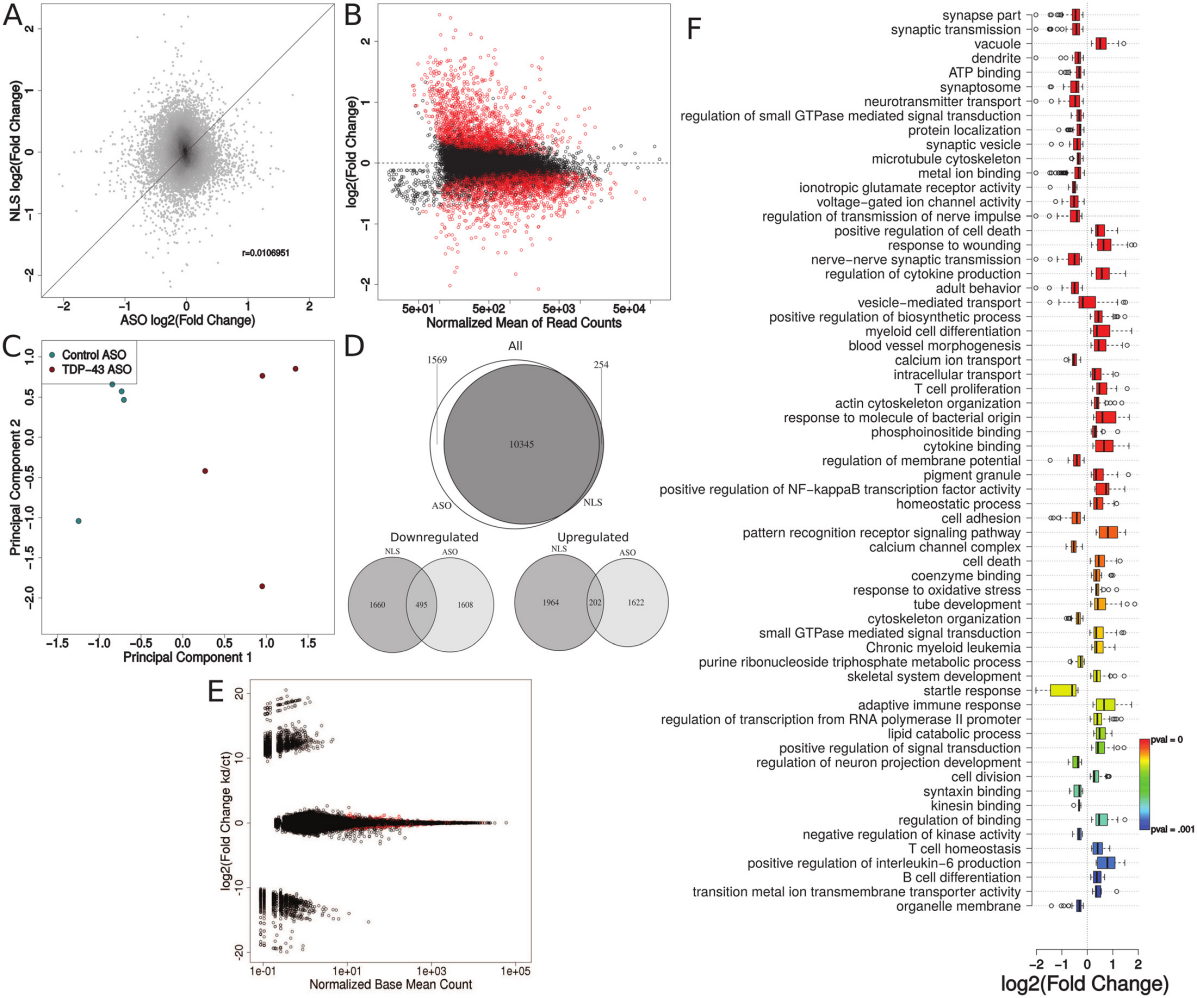
Transcriptomic comparison between ΔNLS-hTDP-43 overexpression and TDP-43 ASO knockdown. (A), Transcriptomic comparison of the 10,345 genes meeting the minimum read count in both experiments shows no correlation between ΔNLS-hTDP expression and TDP-43 ASO knockdown (Pearson r = 0.011). (B), Differential expression analysis reveals that 3,714 out of 11,916 genes meeting the minimum read count threshold were significantly differentially expressed (Benjamini-Hochberg corrected p-value < 0.05, red points) upon ASO knockdown, with no bias of read counts on significance. (C), Multidimensional scaling and principal component analysis shows distinct global transcriptomic profiles for TDP-43 ASO knockdown (n = 4) and control ASO (n = 4) mice. (D), Comparison of genes meeting the minimum read counts in both experiments shows that although the majority of genes are present in both experiments, those that are significantly differentially expressed show very little overlap between ΔNLS-hTDP expression and TDP-43 ASO knockdown. (E), Splicing analysis shows that 1,029 out of 250,620 exons are differentially expressed upon ASO-mediated TDP-43 knockdown. (F), Pathway analysis suggests that most of the transcriptomic effects due to ASO knockdown are associated with the wound response and neuronal death.

We found 11,916 expressed genes in this dataset using the same minimum read count threshold as earlier. We first compared the expression of the 10,345 genes meeting the minimum read count threshold in both datasets (ΔNLS-hTDP-43 versus ASO) to check whether the transcriptomic changes due to ΔNLS-hTDP-43 expression versus ASO knockdown of TDP-43 were generally similar. This analysis demonstrated a complete lack of correlation between the two sets of mice (Pearson correlation coefficient r = 0.011, Fig 3a).

Next, we compared the differentially expressed genes in the two models. Of the 11,916 genes meeting the minimum read threshold in the ASO dataset, analysis with DESeq2 identified 3,714 as significantly differentially expressed, of which 1,903 were upregulated and 1,811 were downregulated upon TDP-43 ASO knockdown (Fig 3b). Our analysis discovered many more genes than the 362 upregulated and 239 downregulated found in the original report, likely due to our improved mapping power as well as the statistical improvements of DESeq2 over the original approach, which used a simple Z score statistic based on the assumption of reads following a normal distribution to detect differential expression [14,20]. Additionally, in the original analysis, samples were merged together according to experimental group (control vs. TDP-43 ASO) before performing differential expression analysis which appeared to result in a loss of statistical power. However, principal component analysis confirmed the clustering of samples within knockdown and control conditions, reflecting true transcriptomic changes due to TDP-43 knockdown (Fig 3c).

Strikingly, although the two datasets shared 10,345 expressed transcripts meeting the minimum read threshold (corresponding to 97.58% of expressed genes in the ΔNLS experiment and 86.81% of expressed genes in the ASO experiment), the two experiments revealed vastly different effects on the expression levels of these transcripts. In fact, only 495 and 202 were significantly down- or up-regulated in both datasets, respectively (Fig 3d). Thus, the effects of overexpressing ΔNLS-hTDP-43 seem to be distinct from those caused by a direct loss of TDP-43 function, again suggesting that many of the transcriptomic changes observed in ΔNLS-hTDP-43 mice may be due to a cytoplasmic gain of TDP-43 function.

We described above that ΔNLS-hTDP-43 mice exhibit a low number of splicing changes. To demonstrate that this was not due to a lack of power to detect splicing changes in our analysis pipeline, we performed an identical DEXSeq analysis on the RNA sequencing data from the TDP-43 ASO knockdown experiment. Remarkably, 1,029 out of 250,620 exons were significantly differentially spliced upon ASO knockdown, corresponding to 752 unique genes (Fig 3e). The previously described splicing analysis of this data found 788 genes containing splicing alterations using a splicing sensitive microarray [27]. Comparison of our results with these results is not straightforward, as DEXSeq only identifies differential exon usage, while splicing microarrays assay other classes of splicing events including alternative transcription start sites and alternative 3’ or 5’ splice sites. In line with this, we found that 115 of the genes we identified as containing changes in exonic splicing events were present in the splicing microarray results. It is important to note that transcriptome wide splicing analysis is still difficult, with different technological platforms and computational methods yielding different results [36]. Despite the low overlap with the splicing microarray analysis, likely due to these technological differences, this analysis nevertheless supports the lack of alternative splicing events due to ΔNLS-hTDP-43 overexpression. Given that the ΔNLS and ASO experiments had similar numbers of uniquely mapped reads, the low number of alternative splicing events identified in the ΔNLS mice does not appear to be due to insufficient read depth or inappropriate choice of methodology. Rather, there appears to be a real paucity of alternative splicing events upon ΔNLS-hTDP-43 overexpression, supporting the hypothesis that the main effects of ΔNLS-hTDP-43 are due to a cytoplasmic gain of function rather than a nuclear loss of function.

Next, to compare the enriched pathways in the two experiments, we performed an identical pathway analysis procedure on the ASO data. In contrast to the 8 significant pathways identified in the ΔNLS-hTDP-43 experiment, we found 64 significantly enriched pathways among the differentially expressed genes (EASE score P-value < 0.001, Fig 3f). These pathways appear to reflect an overall wound response with associated neuronal loss: nearly all of the downregulated pathways represent loss of neuronal function (including synaptic transmission, dendrite, synaptosome, neurotransmitter transport, synaptic vesicle, microtubule cytoskeleton, and many more). Additionally, almost all of the upregulated pathways appear to be related to inflammation and the wound response (including positive regulation of cell death, response to wounding, regulation of cytokine production, myeloid cell differentiation, T cell proliferation, and many more). This indicates that the transcriptomic changes seen in ASO mice may reflect not only the effects of knocking down TDP-43, but also changes due to reaction against the invasive injection of the antisense oligonucleotide [14].

### A Role for TDP-43 in Histone Dysregulation

As chromatin assembly and transcription-related genes were previously found to be dysregulated by microarray [13] and identified again here by RNA sequencing, we sought to further characterize these pathways by investigating whether TDP-43 regulates histone transcripts. It is important to note that the RNA sequencing protocol had a polyadenylation selection step. Histone transcripts can be subdivided into canonical versus variant transcripts: canonical histone transcripts are generally not polyadenylated but rather exhibit a terminal stem-loop structure which is important for their proper trafficking and metabolism. However, misprocessing of canonical histone transcripts can lead to aberrant polyadenylation [37,38]. In contrast, variant histone transcripts are normally polyadenylated [39].

**Fig 4:**
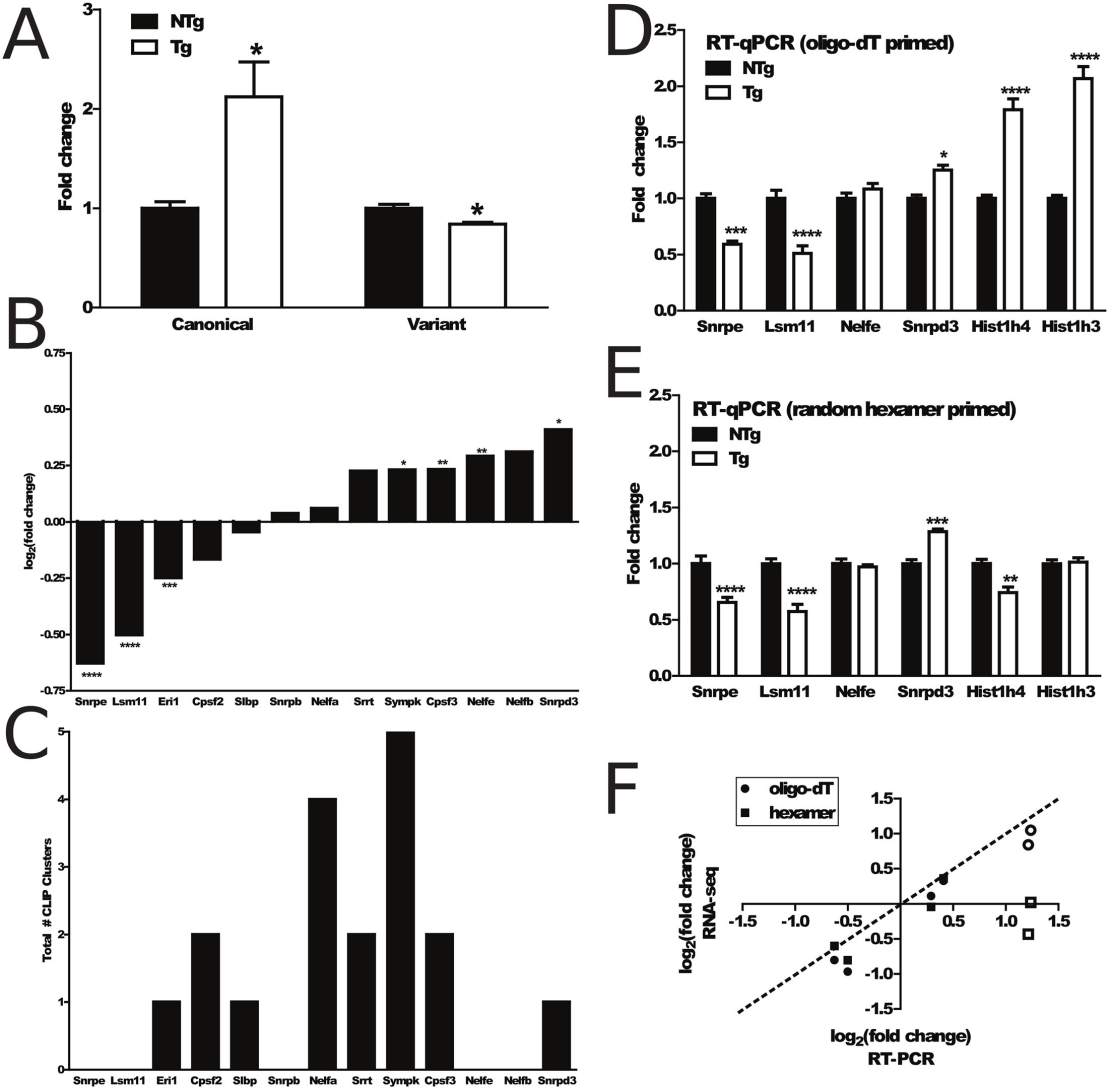
Histone transcript dysregulation in ΔNLS-hTDP-43 mice. (A), Comparison of multimapped read fold change in canonical and variant histones shows a significant upregulation in canonical histones (fold change = 2.12, 2-sided t-test p = 0.046) and significant but slight downregulation in variant histones (fold change = 0.841, 2-sided t-test p = 0.017) in transgenic vs nontransgenic mice. (B), Analysis of log_2_(fold changes) of genes involved in histone 3’ UTR processing reveal an enrichment of significantly changing genes. (C), Analysis of TDP-43 CLIP binding on genes involved in histone 3’ UTR processing shows enrichment for CLIP binding. (D), RT-qPCR validation of histone 3’ UTR processing genes and canonical histones with an oligo-dT selection for polyadenylated RNA confirms the upregulation in aberrantly polyadenylated histone genes in the ΔNLS-TDP-43-expressing mice. (E), Similar RT-qPCR validation with random hexamer priming shows that non-polyadenylated canonical histones are not significantly upregulated in the bigenic mice. (F), Comparison of RNA-seq and RT-PCR analysis validates RNA-seq results, except for histone transcripts (white filled symbols) which revealed distinct results when priming with random hexamers versus oligo-dT’s. *p < 0.05, **p < 0.01, *** p < 0.001, **** p < 0.0001.

We manually generated lists of canonical non-polyadenylated histones as well as histone variants that are polyadenylated in order to compare the effects of the ΔNLS-hTDP-43 overexpression on these types of histone genes (S3 Table). Out of 34 canonical histone genes and 15 variant histone genes, we only found 5 and 10, respectively, that met the read count threshold in our DESeq2 analysis. However, because the 3’ ends of many histone mRNAs are identical and the coding sequence is highly conserved between histone transcripts [40], we reasoned that the DESeq2 analysis, which used only uniquely mapping reads, was limiting our ability to quantify the expression of histone transcripts. With this in mind, we allowed reads to multimap to up to 10 genomic loci within histone genes, weighted by the number of multimappings. Results were normalized against 50 random unchanging genes (see methods). Indeed, although some histone genes still had very low coverage, this analysis retrieved additional coverage for each of the 34 canonical and 15 variant histone genes (S3 Table). Analysis of the difference between bigenic and nontransgenic control histone gene coverage showed that the canonical histones were upregulated in the bigenic mice (fold change = 2.12, 2-sided t-test p = 0.046), while the variant histones were slightly but significantly downregulated in the bigenic mice (fold change = 0.841, 2-sided t-test p = 0.017) (Fig 4a). Notably, the significant upregulation of canonical histones in this poly(A)^+^ selected dataset implies that these transcripts were aberrantly polyadenylated, allowing our protocol to detect them.

To determine whether these changes in canonical histone expression were due to direct interactions with TDP-43, we used High Throughput Sequencing of RNA isolated by Cross-Linking and Immuno-Precipitation (HITS-CLIP) data of TDP-43 RNA binding sites from a previous study to see whether TDP-43 directly binds to canonical histone transcripts [27]. Analysis of the HITS-CLIP data found that out of 28,661 protein coding genes (mm9 refSeq mouse genome), 7,697 genes had TDP-43 binding clusters in their exons, introns, 3’ UTRs, or 5’ UTRs. However, only one canonical histone transcript contained any significant TDP-43 binding sites (*Hist1h2bc* with 2 hits in the 3’ UTR) while three variant histone transcripts contained TDP-43 binding sites (*H2afy*, *H2afy2*, and *H3f3a*), suggesting that the majority of TDP-43’s effects on histone transcripts are due to indirect interactions.

We next hypothesized that aberrant polyadenylation of histone transcripts could be due to a dysregulation of transcripts that regulate histone 3’ UTR processing. We manually generated a list of such genes involved in histone 3’ UTR processing (Table 1). Analysis of this list showed that several of these genes were significantly dysregulated in bigenic mice (Fig 4b). These transcripts were also enriched for TDP-43 RNA binding sites from the HITS-CLIP data (Fig 4c), suggesting a direct role of TDP-43 in regulating these transcripts.

**Table 1:** List of genes involved in histone 3’ UTR processing.

Gene Symbol	Present in DESeq?*	Role
Cpsf2	Yes	Likely to interact with CPSF-73 for cleavage
Cpsf3	Yes	Endonuclease, cleaves histone pre-mRNAs
Eri1	Yes	Aids Slbp in stem loop binding, U7 snRNP stabilization
Lsm10	No	Part of U7 snRNP, process histone mRNA for splicing
Lsm11	Yes	Part of U7 snRNP, process histone mRNA for splicing
Nelfa	Yes	Participates in histone 3’ UTR processing
Nelfb	Yes	Participates in histone 3’ UTR processing
Nelfe	Yes	Participates in histone 3’ UTR processing
Slbp	Yes	Binds stem loop, essential factor
Snrpb	Yes	Part of U7 snRNP, process histone mRNA for splicing
Snrpd3	Yes	Part of U7 snRNP, process histone mRNA for splicing
Snrpe	Yes	Part of U7 snRNP, process histone mRNA for splicing
Snrpf	No	Part of U7 snRNP, process histone mRNA for splicing
Snrpg	No	Part of U7 snRNP, process histone mRNA for splicing
Srrt	Yes	Promotes proper histone 3’ UTR formation
Sympk	Yes	Heat sensitive, essential component of processing machinery
Zfp100	No	Limiting factor in 3’ UTR processing reaction

*Indicates whether this transcript met the minimum threshold count for DESeq2 analysis.

Notably, survival of motor neuron (*Smn*), another protein implicated in motor neuron degeneration, is also known to regulate histone 3’ UTR processing [38]. Interestingly, *Smn* splicing is regulated by TDP-43 [30], and its transcript was significantly downregulated upon ΔNLS-hTDP-43 expression in our dataset (log_2_(fold change) = -0.34, adjusted p-value = 0.001034), further supporting the hypothesis that the transgene overexpression leads to aberrant histone 3’ UTR processing.

To validate these changes in histone 3’ UTR processing genes, we performed RT-qPCR on the same samples used for RNA sequencing to measure canonical histone transcripts (*Hist1h4*, *Hist1h3*), and several transcripts that regulate histone 3’ UTR processing (*Snrpd3*, *Nelfe*, *Snrpe* and *Lsm11*). To test whether the observed changes in canonical histones were due to aberrant polyadenylation, we used both an oligo-dT primer, which selects for polyadenylated RNA transcripts, and random hexamer primers, which have no such selection bias. RT-qPCR measurements using either oligo-dT priming or random hexamer priming validated our RNA-seq data for transcripts which regulate histone 3’ UTR processing (Fig 4d and 4e). Interestingly, in the oligo-dT primed experiment, canonical histone genes were significantly upregulated (*Hist1h4* fold change = 2.07, t-test p = 0.0001; *Hist1h3* fold change = 1.79, t-test p = 0.00003; Fig 4d), while the random hexamer primed experiments revealed no upregulation in these genes (Fig 4e). In fact, *Hist1h4* was significantly downregulated in the bigenic mice when there was no poly(A)^+^ selection step (fold change = 0.74, t-test p = 0.003, Fig 4e). Visualized another way, comparison of the expression changes based on RNA-seq versus RT-qPCR showed a high correlation with the exception of the random hexamer primed canonical histone genes (Fig 4f, white boxes). This confirms that ΔNLS-hTDP-43 expression leads to differential expression of transcripts which regulate histone 3’ UTR processing, coincident with an upregulation in aberrantly polyadenylated canonical histone genes. Additional mechanistic studies are required to establish a direct link between ΔNLS-hTDP-43 overexpression and the observed histone dysregulation, but these results suggest that this dysregulation may be mediated through changes in expression of histone 3’ UTR processing genes.

To confirm that these changes were due to transgene overexpression, we performed RT-qPCR on four groups of mice: bigenic mice maintained on doxycycline until 28 days of age followed by 10 days off doxycycline, bigenic mice maintained on doxycycline until sacrifice, nontransgenic mice maintained on doxycycline until 28 days of age followed by 10 days off doxycycline, and nontransgenic mice maintained on doxycycline until sacrifice (Table 2). First, we confirmed that the levels of polyadenylated histone transcripts (Hist1h3 and Hist1h4, as determined by calculating the amount of oligo-dT primed histone cDNA normalized by the amount of random hexamer primed histone cDNA) were no longer altered in transgenic mice maintained on doxycycline.

**Table 2:** qPCR validation results for control validation groups. Off-dox refers to mice fed a doxycycline-containing diet until 28 days of age followed by 10 days off doxycycline before sacrifice, while on-dox refers to mice fed a doxycycline-containing diet until sacrifice (n = 4 to 5 per group). Values shown as mean ± SE. In top two rows (Hist1h3, Hist1h4), values are ratios of oligo-dT:random hexamer-primed expression, and in remaining rows, values are random hexamer-primed expression values normalized to housekeeping genes.

	Bigenic		Non-transgenic		Two-way ANOVA			Post-hoc analysis	
	Off-dox	On-dox	Off-dox	On-dox	Genotype	Treatment	Interaction	Off-dox	On-dox
**Hist1h3**	2.11 ± 0.18	1.39 ± 0.33	0.88 ± 0.10	1.10 ± 0.05	0.0020	0.7239	0.0102	***	ns
**Hist1h4**	1.45 ± 0.05	1.17 ± 0.09	0.93 ± 0.04	1.07 ± 0.04	0.0006	0.6004	0.0071	***	ns
**Snrpe**	0.79 ± 0.03	1.10 ± 0.05	0.95 ± 0.04	1.04 ± 0.03	0.2326	0.0160	0.0002	*	ns
**Lsm11**	0.68 ± 0.17	0.93 ± 0.10	0.99 ± 0.06	1.01 ± 0.05	0.0779	0.2068	0.2988	ns	ns
**Nelfe**	1.03 ± 0.04	1.05 ± 0.04	0.97 ± 0.03	1.02 ± 0.03	0.2879	0.3460	0.6109	ns	ns
**Snrpd3**	1.28 ± 0.06	1.02 ± 0.06	1.07 ± 0.03	0.94 ± 0.04	0.0127	0.0023	0.2149	*	ns

*p < 0.05,

***p < 0.001.

We also extended these analyses to measure the transcript levels for 3’UTR processing genes. The change in Snrpe and Snrpd3 expression observed in bigenic mice off doxycycline was no longer observed in bigenic mice maintained on doxycycline, and there was a non-significant trend towards the same effect for Lsm11. These results demonstrate that repressing transgene expression with doxycycline reverses the histone and 3’UTR processing gene dysregulation in bigenic mice.

### Alteration of Nuclear Morphology

**Fig 5:**
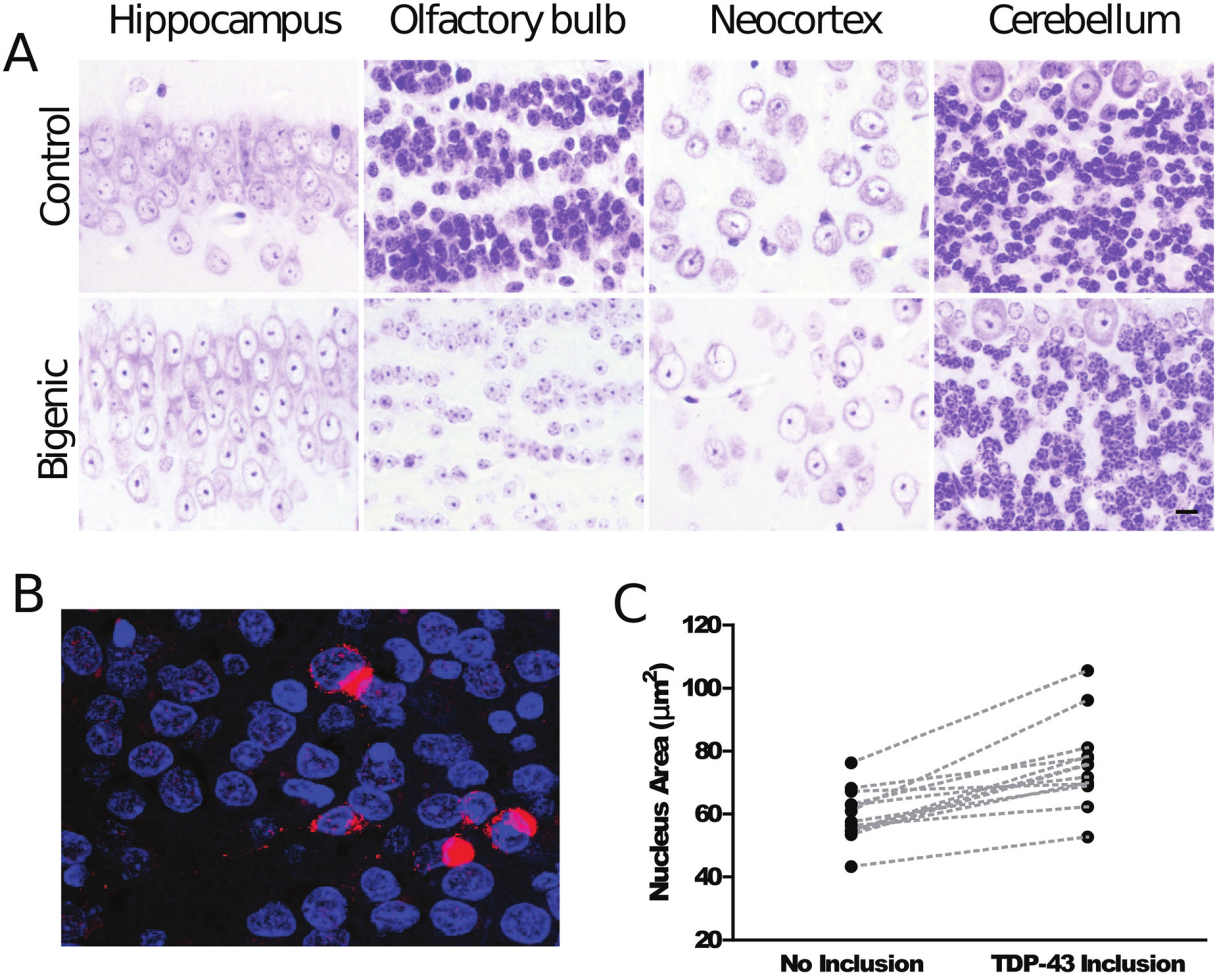
Altered nuclear morphology associated with cytoplasmic TDP-43 protein. (A) Cresyl violet staining of bigenic mice brain sections reveals abnormal nucleolar and chromatin structure and nucleomegaly affecting hippocampal pyramidal (CA1) neurons, olfactory bulb granular neurons, and neocortical neurons. Cerebellar granular and Purkinje neurons, where the transgene is not expressed, show no nuclear morphologic change. (B) Representative confocal immunofluorescence image of human hippocampal dentate gyrus neuronal nuclei stained with DAPI (blue) with and without TDP-43 inclusions (red). (C) Quantification of average cross sectional nuclear area of neurons without TDP-43 inclusions (n = 254) and neurons with TDP-43 inclusions (n = 133) from 13 different FTLD-TDP cases. Median value of nuclear area for neurons without TDP-43 inclusions is 57.56 μm^2^ and is 75.66 μm^2^ for neurons with TDP-43 inclusions. Paired t-test reveals a significant difference in nuclear size (p < 0.0001).

Given that ΔNLS-hTDP-43 expression leads to dysregulation of transcription, chromatin assembly, and nucleolar function together with apparent changes in histone transcript processing, we next investigated the effects of ΔNLS-hTDP-43 overexpression on both chromatin and nucleolar structure. Histological analysis of the ΔNLS-hTDP-43 expressing mouse brain sections stained with cresyl violet revealed widespread nuclear abnormalities (Fig 5a). Normal pyramidal neurons within the hippocampus demonstrate moderately sized nucleoli together with several additional foci of heterochromatin (n = 16 nontransgenic or monogenic mice). In contrast, hippocampal pyramidal neurons from bigenic mice demonstrated markedly enlarged nucleoli. Moreover, the nuclear chromatin was altered such that there was prominent, densely packed perinucleolar chromatin and a loss of non-nucleolar heterochromatin. These changes were accompanied by uniform enlargement of nuclei (i.e. nucleomegaly). These morphologic changes were seen not only in hippocampal pyramidal neurons, but also in many neuronal populations which express the TDP-43 transgene in this transgenic model including neocortical neurons, olfactory bulb granular neurons and others (Fig 5a). In contrast, regions devoid of transgene expression such as the cerebellum did not exhibit histologic evidence of chromatin abnormalities or nucleomegaly (Fig 5a). These histologic changes were seen in all bigenic mice in which transgene expression was turned on for 3 months or longer (n = 14, ranging from 3 months to 15 months of transgene expression). In contrast, nuclear changes were subtle or absent in mice with shorter lengths of transgene expression (n = 21, ranging from 0 to 2 months of transgene expression). Overall, these results corroborate the results of the transcriptomic analysis, supporting a role of cytoplasmic TDP-43 overexpression in chromatin assembly pathways as well as nucleolar structure and function.

To extend these observed effects on nuclear structure, hippocampal sections from human cases of FTLD-TDP were immunostained for TDP-43 protein and examined for changes in nuclear structure. Hippocampal dentate gyrus granular neurons were studied given their uniform size and shape. Although overt chromatin abnormalities were not obvious, nuclei of neurons with cytoplasmic TDP-43 aggregates appeared to be slightly larger than nuclei from adjacent uninvolved neurons. To verify this finding, the cross-sectional areas of neuronal nuclei with and without TDP-43 inclusions were measured from three-dimensional confocal microscopy image stacks. 133 nuclei associated with TDP-43 inclusions and 254 adjacent uninvolved nuclei were measured from 13 human cases which revealed that nuclei of neurons with cytoplasmic TDP-43 aggregates had a 31.4% larger cross-sectional area (75.66 μm^2^) relative to nuclei of adjacent uninvolved neurons (57.56 μm^2^) (Fig 5b). Comparing the average cross sectional area of nuclei from neurons with inclusions against those without by case showed that this difference was significant (paired t-test p < 0.0001, Fig 5c). Thus, nucleomegaly is a feature of both experimental models of TDP-43 proteinopathy and human disease neurons.

## Discussion

The integrative analysis of the molecular phenotype of the ΔNLS-hTDP-43 expressing mouse performed in this study revealed varied effects of the cytoplasmic overexpression of TDP-43. Transcriptomic analysis using RNA sequencing technology revealed broad effects of the transgene overexpression, including upregulation of genes related to transcription, chromatin assembly, the nucleolus and protein metabolism, as well as downregulation of genes related to synaptic activity and posttranslational modification. We identified specific dysregulation in histone 3’ UTR processing genes due to cytoplasmic TDP-43 overexpression, coincident with the surprising overrepresentation of canonical histone transcripts in the poly(A)^+^ selected RNA sequencing data and elucidating a specific role for TDP-43 in the previously reported dysfunction of the chromatin assembly pathway [13]. Defects in chromatin assembly and the nucleolus were also confirmed histologically where abnormal patterns of chromatin staining and nucleomegaly were observed. Importantly, these abnormal patterns were identified in both the experimental mouse model as well as in human FTD-TDP patients, suggesting that the transcriptomic changes underlying these chromatin assembly defects may also be relevant to the disease in humans. Nucleomegaly has not been well studied in neurodegenerative diseases, but it has been observed in asymptomatic Alzheimer’s disease patients [41].

In the broader context of TDP-43 biology, this analysis suggests that a gain of cytoplasmic TDP-43 function affects nuclear dynamics. Specifically, the presence of increased cytoplasmic levels of TDP-43 protein leads to aberrations in histone 3’ UTR processing gene transcription, resulting in defects in histone processing such as irregular polyadenylation that contribute to defects in the chromatin assembly process. Several lines of evidence support this claim, including the presence of aberrantly polyadenylated canonical histone transcripts in the RNA sequencing data, PCR validation of increases in polyadenylated canonical histones, and histological validation of defects in chromatin assembly.

Although we had previously hypothesized that the toxic effects of the ΔNLS-hTDP-43 overexpression were caused by dysregulation of endogenous mTDP-43, therefore mainly representing a loss of function [13], our comparison with the ASO TDP-43 knockdown experiment showed that, at least on the transcriptomic level, mice expressing ΔNLS-hTDP-43 show little evidence of a pure loss of function. It is clear from our previous study that the endogenous nuclear mTDP-43 levels are reduced following ΔNLS-hTDP-43 expression, but hTDP-43 was also shown to be present at significant levels in the nucleus [13]. Our fractionation protocol confirmed that the levels of total (mouse and human) TDP-43 protein were similar in nuclei of both bigenic and nontransgenic mice, suggesting that despite the introduction of a mutation within the nuclear localization signal, the transgene is able to generate enough nuclear hTDP-43 protein to compensate for the downregulation in mTDP-43 expression.

The lack of transcriptomic similarity with the ASO knockdown, the unchanged level of nuclear total TDP-43 protein, and the marked increase in cytoplasmic TDP-43 protein levels altogether suggest that the widespread degeneration and transcriptomic changes observed in the ΔNLS-hTDP-43 expressing mice may be mainly due to a toxic gain of cytoplasmic TDP-43 function. This is further supported by the paucity of splicing changes upon expression of ΔNLS-hTDP-43, implying that the exogenous hTDP-43 seems to be able to mostly compensate for the disruption of endogenous mTDP-43 nuclear splicing regulatory activity. It is important to note that cytoplasmic TDP-43 inclusions are very rare in these mice, suggesting that the effects observed in this model seem to be due specifically to a gain of cytoplasmic function as opposed to toxic aggregation. Furthermore, comparison of the transcriptomic results with HITS-CLIP data of TDP-43 RNA binding partners showed that six out of the eight significantly changing pathways were significantly enriched for TDP-43 binding targets (data not shown). This suggests that many of the transcriptomic effects in these mice involve the direct interactions of TDP-43 with its RNA targets, as opposed to indirect effects arising from the cytoplasmic mislocalization.

This analysis also confirmed our previous report of ΔNLS-hTDP-43 affecting chromatin assembly genes. Our RNA-seq, HITS-CLIP and RT-PCR analyses showed that TDP-43 interacts with and alters the expression of several transcripts involved in histone 3’ UTR processing, suggesting that canonical histone dysregulation may be a mechanism by which chromatin assembly pathways and nuclear dynamics are disrupted. These transcriptomic results were supported by our histological findings which showed irregular chromatin structure in neurons expressing ΔNLS-hTDP-43.

Aberrant histone transcript polyadenylation has been shown to affect translation of histone transcripts. Knockdown of stem loop binding protein, the protein that binds the stem loop of histones and facilitates proper histone transcript processing and trafficking, leads to aberrantly polyadenylated histone transcripts which are retained in the nucleus and therefore not translated [42]. However, another study suggested that polyadenylated histone transcripts can be transported to the cytoplasm, bound to polyribosomes, and translated [43]. Furthermore, as histone translation is highly dependent on cell cycle stage [39], the regulation of histone transcripts in non-dividing neurons is not well understood. We did not detect a change in the levels of histone protein H3 in bigenic mice compared to nontransgenic controls at steady state (Fig 2). Given the strong physical association between histone proteins and chromatin, a reduction in histone protein synthesis may not lead to changes in steady state histone levels but rather could lead to more subtle changes in histone turnover or flux. Another layer of complexity is that independent of any effects on translation, the degradation of these histone transcripts, which is also cell cycle dependent [39], may also be affected by aberrant polyadenylation.

Together, this study further supports the importance of the role of TDP-43 in transcriptional regulation and identifies a specific effect of the overexpression of cytoplasmic TDP-43 on nuclear dynamics and misprocessing of the 3’ end of histone transcripts. Although this model artificially induces a gain of cytoplasmic function, the results observed here provide an insight into the consequences of pathological cytoplasmic accumulation of TDP-43. The question of whether the pathological effects of TDP-43 in ALS and FTLD-TDP are caused by a nuclear loss of function or a cytoplasmic gain of function is still uncertain [44], but this study provides an improved understanding of the effects of a cytoplasmic gain of function. The mice expressing the ΔNLS-hTDP-43 transgene suffered neurodegeneration without common cytoplasmic TDP-43 aggregations [13], suggesting that the mechanisms identified in this study may play an important role in the pathological process even without the development of TDP-43 inclusions, perhaps modeling a pre-aggregated disease state.

## Supporting Information

S1 Table Alternatively spliced genes in ΔNLS-hTDP-43 mice. Exon IDs include 3 digits because some annotated genes include hundreds of exons. NA gene symbols represent exons from unannotated genes, and both unannotated genes and exon IDs are directly from the Ensembl mm9 genome annotation [23].
(XLSX)

S2 Table RT-qPCR Primer Sequences. (XLSX)

S3 Table Canonical and variant histone genes in the multimapping analysis. Entries with the same gene cluster entry represent different members of highly similar clusters. Type c = canonical, v = variant.
(XLSX)
